# Association of Dietary Patterns and Pre-pregnancy Body Mass Indices With Gestational and Birth Outcomes in Pregnant Emirati Women: A Cross-Sectional Study

**DOI:** 10.7759/cureus.75038

**Published:** 2024-12-03

**Authors:** Lolowa Almekhaini, Shamsa A. Awar, Sania Al Hamad, Fatmah Almesmari, Maha Khaled, Nehaya Qasem, Fatima Bahwan, Elhadi H Aburawi, Hassib Narchi

**Affiliations:** 1 Pediatrics, United Arab Emirates University, Al Ain, ARE; 2 Obstetrics and Gynecology, United Arab Emirates University, Al Ain, ARE; 3 Obstetrics and Gynecology, Mediclinic Middle East, Dubai, ARE; 4 Obstetrics and Gynecology, Al Wagan and Tawam Hospitals, Al Ain, ARE

**Keywords:** diet, neonatal outcome, obesity, pregnancy, united arab emirates

## Abstract

Background and aim: This cross-sectional, community-based study examined the association of dietary intake of pregnant Emirati women and their pre-pregnancy body mass index (pBMI) with maternal and neonatal outcomes.

Methods: The study was conducted at tertiary hospitals in Abu Dhabi, United Arab Emirates, where 323 pregnant women reported their weekly dietary intake using the Arabic version of the food frequency questionnaire. Dietary patterns (DPs) were established using factor analysis of consumed foods followed by cluster analysis. Maternal pBMI was recorded within three months of the current pregnancy.

Results: Three hundred and twenty-three pregnant women were enrolled, with a median age of 28.6 years (range: 18-35). A high proportion were overweight (n=109, 34%) and 20.9% (n=67) were obese. Data was available for 306 infants who had a median gestational age of 38 weeks (range: 25-42), and the majority were full-term (n=255, 89.8%). The median birth weight was 3035 grams (range: 850-4185) with nine (3.8%) being small for gestational age. There were two distinct groups of maternal DPs: "natural ingredients" and "processed foods". There was no statistically significant association between DPs and maternal characteristics nor with their infants' characteristics. None of the maternal factors was significantly associated with the mode of delivery or maternal complication. Only maternal age was significantly associated with the one-minute Apgar score and the duration of neonatal stay in the hospital, while pre-pregnancy weight was significantly associated with neonatal weight Z-score, neonatal complications, and admission to the neonatal intensive care unit.

Conclusion: We found no significant difference in DPs among maternal pBMI groups nor in pregnancy or neonatal outcomes, possibly related to unmeasured confounders, such as maternal exercise, detailed quantitative and qualitative analysis of macronutrient and micronutrient intake, and socioeconomic, genetic, or environmental factors. With the increasing rate of obesity and the changes in the dietary habits in our population, a periodical review of their resulting impact on pregnancy and neonatal outcomes is required to inform public health policies.

## Introduction

The nutritional status and eating habits of pregnant women during pregnancy significantly influence maternal health and birth outcomes [1-9]. The accelerating pace of economic and nutritional change has driven a global surge in unhealthy eating behaviors. This shift has far-reaching implications for public health, contributing to a substantial disease burden [8,10-12]. Globally, the prevalence of overweight and obesity, especially among the young population, has increased due to the increased consumption of high-calorie meals; this was observed too in the United Arab Emirates (UAE) [8,13].

The increased prevalence of obesity in females of reproductive age is associated with maternal complications such as preeclampsia, gestational diabetes mellitus, atherosclerosis, preterm delivery, low birth weight, and small or large for gestational age infants and contributes negatively to the maintenance of exclusive breastfeeding after birth [1,2,5-8,10,11,14,15]. Moreover, it has also been shown that it also has an impact on the neurodevelopment of the offspring [16]. Despite the availability of dietary guidelines during pregnancy, it was demonstrated that they are not being met, especially during the initial stages of pregnancy [17-20]. Diet myths and limited knowledge might be possible causes and are also influenced by sociodemographic variables, economic factors, living with children, attitudes and beliefs, prior, family support, and lack of appreciation expectations of nutrition outcomes [21-24]. In the UAE, the prevalence of obesity is increasing, and dietary habits and their association with maternal health and infant outcomes still need to be quantified [12,25]. Thus, conducting culture-based studies on the eating habits and dietary patterns (DPs) of pregnant women in the UAE is imperative. Their impact on fetal health would be instrumental in improving maternal educational guidance and counseling with a view to minimize maternal and infant complications. However, the DP is strongly dependent on traditions and the social context in any culture, thus depending on both human and environmental factors. Thus, a culturally validated survey is needed to assess the dietary habits of pregnant women in the UAE to accurately represent their DPs [6,12]. In this study, we used a culture-specific questionnaire that has already been validated in our population outcome. The aim is to study the correlation between the DPs of pregnant Emirati women and their pre-pregnancy body mass index (pBMI) with maternal and neonatal outcomes.

## Materials and methods

Study design and population

This study is a cross-sectional, community-based sample evaluated with a culture-specific non-quantitative food frequency questionnaire (FFQ) which was developed and validated in a previous study in the UAE to assess the DPs of pregnant women (Appendix A and Appendix B) [26]. The FFQ was completed by Emirati pregnant mothers in the presence of a trained staff during the mothers' visit to the obstetric clinic for routine antenatal healthcare at two tertiary hospitals in Al Ain, namely, Al Wagan and Tawam Hospitals, between June 2019 and May 2022. The antenatal care program provides an educational program on maternal nutrition and guidance on physical activity, as recommended in the international guidelines of the National Institute for Health and Care Excellence (NICE) and the American College of Obstetricians and Gynecologists (ACOG).

Data collected

The participant baseline information included the following: the first day of their last menstrual period (LMP) and its certainty, the regularity and duration of the menstrual cycle, participant age, educational level, parity, pBMI, and complications during delivery. We enrolled pregnant Emirati women within the age range of 18-35 years, with a live singleton pregnancy. The delivery due date was confirmed by fetal ultrasound at 12-13 weeks of gestation. The mean pre-pregnancy weight (kg) and height (cm) of participants were collected from their medical records within the three months preceding the current pregnancy. pBMI (weight (kg)/height² (m²)) was classified into four groups, according to the US Centers for Disease Control and Prevention (CDC), National Institutes of Health (NIH), and WHO criteria: underweight (<18.5 kg/m^2^), normal weight (18.5-24.9 kg/m^2^), overweight (25-29.9 kg/m^2^), and obese (≥30 kg/m^2^) [27]. Women were excluded from the study if the first ultrasound at 12 weeks of gestation showed multiple pregnancy, fetal anomaly, miscarriage, or fetal death. Women who were current smokers and diagnosed with chronic diseases or autoimmune disorders, any type of cancer, infection with the human immunodeficiency virus, or pregnancy-associated complications such as gestational diabetes or preeclampsia were excluded.

Data collected included maternal age, educational level, parity, pBMI, and complications during delivery. Perinatal and birth outcomes were assessed and included perinatal death, birth gestational age, sex, birth weight, head circumference, length and their respective Z-scores, mode of delivery, Apgar scores, neonatal complications (e.g., hypoglycemia, hyperbilirubinemia, birth trauma, respiratory distress syndrome, cardiac problem, congenital malformations, infection and seizures, admission to intensive care, duration of hospitalization), and prevalence of breastfeeding on discharge home.

Dietary assessment

We used a culture-specific non-quantitative FFQ (Appendix A and Appendix B) [25]. It was subdivided into 13 categories of food groups: dairy foods (eight items), composite dishes (11 items), proteins (17 items), vegetables (15 items), cereals, pasta, and potatoes (eight items), sandwiches and baked snacks (six items), bread and savory biscuits (nine items), spreads on breads, on vegetables, or added on salads or drinks (15 items), fruits and dried fruits (12 items), beverages (seven items), sweets and snacks (19 items), and soups (three items). There was a total of 140 food items (Appendix C). The consumption frequency included once/week, 1-3 times/month, 2-3 times/day, 2-4 times/week, 4-5 times or more/day, 5-6 times/week, 6 + times/day, never or less than once/month, and once/day. For all analyses, we converted all dietary data to food consumption frequency per week.

Identification of DPs

Twelve food families with related nutritional characteristics were compiled from the initial pool of the items present in the FFQ. Factorial and cluster analyses were used to identify the DPs of pregnant women. The participants were divided into distinct DPs generated according to two steps: First, a factorial analysis of the 13 categories of food groups was conducted based on the principal component analysis (PCA) technique with Kaiser normalization after the appropriate checking of assumptions and sample adequacy, followed by a Varimax orthogonal rotation. It identified the inter-correlations between the food groups as the factors of foods consumed with identical frequencies on an individual level. A scree plot of PCA results was constructed with the x-axis representing the principal components and the y-axis showing the corresponding eigenvalues. The elbow point of the graph (where the slope of the scree plot changes abruptly) corresponds to the eigenvalue of 1.0 (Kaiser criterion), which indicates the optimal number of components to retain.

Subsequently, the above factors were included in a cluster analysis which was used to group the participants into different groups based on similar frequency patterns in food consumption, using the K-means clustering method. The analysis resulted in two distinct DPs, which were named and used as DPs in the bivariate and multivariable analyses. Factor scores are standard scores with a mean of 0 and are estimates of underlying latent constructs. Eigenvalues are the weights in a linear transformation when computing principal component scores and indicate the amount of variance explained by each principal component or each factor.

For missing values, we used a multiple imputation method which automatically adapts to the missing values in the analysis. The initial dataset with missing data was used for all descriptive univariate analyses, and the expanded dataset by multiple imputation method was used for all multivariate analyses.

After checking for normality of the distribution, continuous variables were represented as mean±standard deviation (SD) if normally distributed or median and interquartile range (IQR) if skewed and categorical variables as number and percentage. 

For bivariate analysis, we used the two-sample t-test to compare the means of continuous variables between the two groups, after checking for normality. To compare proportions or percentages, we used the chi-squared test or Fisher's exact test when the results were less than five. Finally, for multivariate analysis to adjust for possible confounders, we only included the variables with a p-value <0.10 in the univariate analysis in a general linear model (GLM), using the Gaussian and the binomial families for continuous and nominal outcome variables, respectively. All analyses were performed with the Stata Version 17 (StataCorp LLC, College Station, Texas, United States), and results were considered significant at a p-value <0.05.

Ethical approval

Approval to conduct the study was obtained from the Tawam Human Research Ethics Committee (approval number: THREC-627). The study was funded by the College of Medicine and Health Sciences, United Arab Emirates University, Al Ain, United Arab Emirates (grant application number: NP-19-1).

## Results

Maternal characteristics

The median age of enrolled women was 28.6 years (range: 18-35). A high proportion were overweight (n=109, 34%) and 20.9% (n=67) were obese. The majority (n=299, 98%) were either high school graduates or university undergraduates. The details are shown in Table 1.

**Table 1:** Maternal characteristics (original dataset) Continuous variables were represented as mean±SD when normally distributed and categorical variables as number and %. Some missing values occurred in the variables pBMI: pre-pregnancy body mass index; SD: standard deviation; %: percentage

Variable	Results
Number of participants	323
Age (years): mean±SD	28±6
Pre-pregnancy weight (kg): mean±SD	68.9±18.4
pBMI (kg/m^2^): mean±SD	27.5±7.3
Extremely obese: number (%)	40 (12.5%)
Obese: number (%)	67 (20.9%)
Overweight: number (%)	109 (34%)
Normal: number (%)	99 (30.8%)
Underweight: number (%)	6 (1.9%)
Postgraduate (master's degree or PhD): number (%)	9 (2.9%)
University degree (undergraduate): number (%)	150 (48.5%)
High school: number (%)	140 (45.3%)
Less than high school: number (%)	10 (3.2%)

Neonatal characteristics

Data was available for 306 infants as some mothers delivered in other hospitals. Their median gestational age was 38 weeks (range: 25-42), and the majority of infants were full-term (n=255, 89.8%). The median birth weight was 3035 grams (range: 850-4185) with nine (3.8%) being small for gestational age. The details are shown in Table 2. All infants were discharged home breastfed and no death occurred.

**Table 2:** Neonatal characteristics (original dataset) Continuous variables are represented as median and IQR as they were skewed and categorical variables as number and % NICU: neonatal intensive care unit; IQR: interquartile range; %: percentage

Variable	Value
Number of infants	306
Gestational age (weeks): mean±SD	38±2
Term birth: number (%)	255 (89.8%)
Females: number (%)	151 (49.2%)
Weight (grams): mean±SD	3035±531
Weight (Z-score): mean±SD	-0.35±0.9
Height (cm): mean±SD	50±3.4
Height (Z-score): mean±SD	0.31±1.0
Head circumference (cm): mean±SD	34±1.6
Head circumference (Z-score): mean±SD	0.05±1.09
Small for gestational age: number (%)	9 (3.8%)
Apgar score at one minute: mean±SD	8±1
Apgar score at five minutes: mean±SD	9±0.7
Transfer to NICU: number (%)	6 (2.2%)
Length of hospitalization (days): mean±SD	4.3±2

Dietary analysis

Food Categories and Consumption Patterns

**Figure 1:**
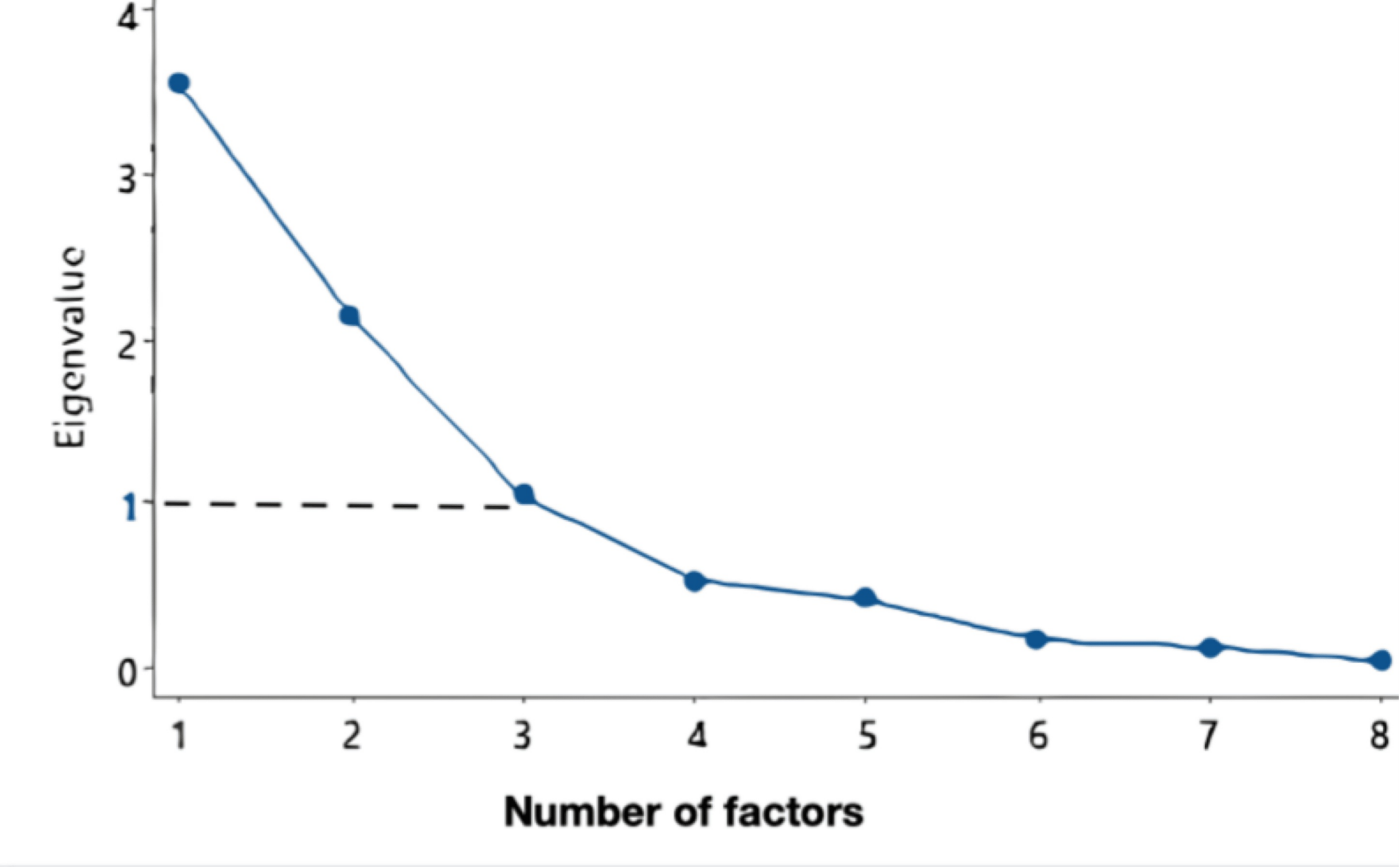
Scree plot of PCA results The x-axis represents the principal components and the y-axis shows the corresponding eigenvalues which indicate the amount of variance explained by each principal component factor. The inflection (elbow) point of the plot (where the slope of the plot curve changes abruptly) corresponds to the eigenvalue of 1.0 (Kaiser criterion), which indicates the optimal number of components to retain. It corresponds in this plot to factor 3, with all factors to the left of it being retained for analysis PCA: principal component analysis

In the factorial analysis used to distinguish the inter-correlations between the food groups, we retained only the factors with an eigenvalue >1 and with a factor loading >+4.0. The scree plot identified three factors (factors 1, 2, and 3) with an eigenvalue >1 explaining 27% of the cases (Figure 1, Appendix D). As factors 4, 5, and 6 had an eigenvalue <1, they were not retained in the analysis of DPs. The Kaiser-Meyer-Olkin (KMO) value of 0.50 (p<0.001 for Bartlett’s test of sphericity) confirmed the adequacy of the sample for the analysis.

Factor 1 (eigenvalue 3.5, explaining 16.3% of the cases): There is high loading on skimmed milk, laban (yogurt), halloumi cheese, stuffed courgettes and cabbage, chicken with rice, luncheon meat, hot dogs, sausages, carrots, broccoli, cauliflower, cabbage, kale, okra, sweet corn, mixed vegetables, cornflakes, rice crispies, sugar puffs, cocoa pops, whole grain cereals, oats, pizza, falafel, sambosa, white bread, Arabic bread, chebab bread, samoon bread, spreads (labneh, cheese), olives, evaporated milk, jam, date molasses, kiwi, plums, peaches, fruit cocktail with added sugar, energy drinks, milkshakes, fruit-flavored drinks, salted sunflower seeds, mixed nuts, um ali (pudding), kunafah, balaleet, lgeimat, qurs, baklawa, maamoul, pudding, biscuit cookies, sponge cake, donuts, cream cakes, chocolate bars, candies, and popcorn.

Factor 2 (eigenvalue 2.2, explaining 5.18%): There is high loading on apples, pears, bananas, oranges, tangerines, cherries, pomegranates, grapes, mangoes, and watermelon.

Factor 3 (eigenvalue 1.0, explaining 4.18% of the cases): There is high loading on cooked or grilled meat or chicken with bread or rice.

DP Assessment

The cluster analysis (Table 3) identified two distinct DPs.

**Table 3:** DP: cluster analysis of factors of consumed food groups Food consumption frequency per week is expressed as number (%). The factor scores are estimates of underlying latent constructs and are expressed as mean±SD *Comparison with the unpaired t-test §Factors 4, 5, and 6 had an eigenvalue< 1 and were therefore not retained for the analysis of DPs DP: dietary pattern; SD: standard deviation; %: percentage

	DP groups		P-value*
	DP 1 (natural ingredients)	DP 2 (processed foods)	
Number (%)	60 (42.9%)	80 (57.1%)	
Scores for factor 1	-0.570±1.016	0.416±0.765	<0.001
Scores for factor 2	0.42±1.152	-0.287±0.753	<0.001
Scores for factor 3	0.020±1.116	-0.031±0.902	0.766
Scores for factor 4^§^	0.231±1.154	-0.154±0.852	0.025
Scores for factor 5^§^	-0.168±1.128	0.121±0.868	0.089
Scores for factor 6^§^	-0.120±0.925	0.080±1.039	0.241

DP 1 (consumption of more foods from factor 2 (p<0.001) and factor 3 (p=0.73)): DP 1 could be described as natural ingredients: fruits, vegetables, legumes, meat or chicken with bread and rice, hares (meat or chicken with boiled barley), arsiya (paste of chicken and rice with a fruity sauce), falafel, whole grains, fish and seafood, olive oil, nuts, seeds, and fat-free or low-fat dairy.

DP 2 (consumption of more foods from factor 1 (p<0.001)): DP 2 could be described as processed foods: dairy, meat, processed meat and poultry, cereals, sweets, salted biscuits, vegetable or legume soup, shawarma, hamburger, fast foods, added sugars, refined grains, sweets, desserts, snack foods, soda, and sugar-sweetened beverages.

These DPs and their correlations with nutrient intakes are shown in Appendix E.

Correlations of DPs With Maternal and Neonatal Characteristics

There was no statistically significant association between the DPs and maternal characteristics (Table 4) nor with their infants' characteristics (Table 5).

**Table 4:** Maternal characteristics and DPs (original dataset) Continuous variables were represented as mean±SD if normally distributed or median and IQR if skewed and categorical variables as number and %. Continuous variables were skewed and were compared with unpaired Student's t-test if normally distributed, the Kruskal-Wallis test if skewed, and the categorical variables with the chi-squared test or Fisher's exact test for small numbers. As missing values were present in some variables and also in the DP classification, the total numbers in the column for all infants are not always equal to the total of DP 1 and DP 2 DP: dietary pattern; pBMI: pre-pregnancy body mass index; PET: preeclamptic toxemia; SD: standard deviation; %: percentage; IQR: interquartile range; *: missing values

	DP groups		P-value
	DP 1 (natural ingredients)*	DP 2 (processed foods)*	
Number of food items*	80 (57.1%)	60 (42.9%)	0.183
Age (years)	28 (7.5)	27.5 (5.5)	0.698
Pre-pregnancy weight (kg)	68 (19.6)	68.4 (22)	1.000
pBMI (kg/m^2^)	26.5 (8.2)	27.4 (8.3)	0.804
pBMI classification			
Extremely obese	10 (12.7%)	8 (13.3%)	0.675
Normal	31 (39.2%)	19 (31.7%)	
Obese	15 (19%)	13 (21.7%)	
Overweight	22 (27.8%)	17 (28.3%)	
Underweight	1 (1.3%)	3 (5%)	
Mother education			
High school	22 (28.6%)	25 (43.1%)	0.092
Less than high school	1 (1.3%)	2 (3.4%)	
Postgraduate (master's degree or PhD)	4 (5.2%)	0 (0%)	
University degree (undergraduate)	50 (64.9%)	31 (53.4%)	
Mode of delivery			
Cesarean section	23 (30.3%)	21 (36.2%)	0.468
Normal vaginal delivery	53 (69.7%)	37 (63.8%)	
Maternal complications	6 (7.5%)	3 (5%)	0.382
Severe PET	0 (0%)	0 (0%)	
Bleeding	1 (1.33%)	0 (0%)	
Bladder injury	0 (0%)	0 (0%)	

**Table 5:** Neonatal characteristics and maternal DP (original dataset) Continuous variables were represented as mean±SD if normally distributed or median and IQR if skewed and categorical variables as number and %. Continuous variables were compared with unpaired Student's t-test if normally distributed, the skewed continuous variables with the Kruskal-Wallis test, and the categorical variables with the chi-squared test or Fisher's exact test for small numbers. As missing values were present in some variables and in the DP classification, the total numbers in the column for all infants are not equal to the total of DP 1 and DP 2 NICU: neonatal intensive care unit; DP: dietary pattern; SD: standard deviation; IQR: interquartile range; %: percentage; *: missing values

	Maternal DP groups		P-value
	DP 1 (natural ingredients)*	DP 2 (processed foods)*	
Number of food items	80	60	
Gestational age (weeks)	39 (2)	39 (1)	0.611
Term birth	67 (93.1%)	48 (92.3%)	0.874
Females	41 (53.2%)	26 (44.8%)	0.333
Weight (grams)	3150 (717.5)	3070 (520)	0.427
Weight (Z-score)	-0.480±0.95	-0.295±1.050	0.640
Height (cm)	50±4	51±4	0.654
Height (Z-score)	0.190±1.13	0.455±1.37	0.274
Head circumference (cm)	34±2	34±2	0.619
Head circumference (Z-score)	-0.130±1.35	-0.100±1.22	0.769
Small for gestational age	2 (3.1%)	1 (2.1%)	0.735
Apgar score at one minute	9 (1)	9 (1)	0.931
Apgar score at five minutes	10 (1)	10 (1)	0.407
Newborn complications	2 (2.5%)	1 (1.7%)	0.736
Fetal distress	1 (1.2%)	0 (0%)	
Hyperbilirubinemia requiring phototherapy	0 (0%)	1 (1.6%)	
Respiratory distress	1 (1.2%)	0 (0%)	
Transfer to the NICU	0 (0%)	3 (5.3%)	0.238
Length of hospitalization (days)	2 (2)	2 (2)	0.727

Univariate Association of Maternal Factors With Pregnancy and Neonatal Outcomes

In the univariate analysis (Table 6), there was a significant association (p<0.10) only between the mode of delivery and the pre-pregnancy weight and between maternal complications and the pBMI classification. Maternal age was significantly associated (p<0.10) with the gestational age at delivery, the Apgar score at one minute, and the duration of neonatal hospital stay. Pre-pregnancy weight was significantly associated with neonatal weight and head circumference Z-score, neonatal complications, and admission to the neonatal intensive care unit. Maternal education level was associated with head circumference Z-score, but the maternal dietary factors showed no association with any neonatal outcomes (Table 7 and Table 8).

**Table 6:** Univariate analysis of maternal factors with pregnancy outcomes (imputed dataset) Comparisons were made with a generalized linear model with the Gaussian family for continuous variables and binomial for binary variables. Results are expressed as coefficients for each variable obtained in the univariate regression model. P-values <0.10 were included in the multivariate model pBMI: pre-pregnancy body mass index; DPs: dietary patterns; CI: confidence interval

	Maternal and pregnancy outcomes			
	Mode of delivery		Maternal complications	
Risk factors	Coefficient (95% CI)	P-value	Coefficient (95% CI)	P-value
Age (years)	0.014 (-0.041, 0.070)	0.612	-0.034 (-0.123, 0.055)	0.453
Pre-pregnancy weight	0.016 (0.0010, 0.032)	0.037	0.020 (-0.002, 0.044)	0.081
pBMI classification	0.477 (-0.074, 1.029)	0.090	0.512 (-0.427, 1.452)	0.286
Educational level	0.346 (-0.151, 0.844)	0.172	-0.145 (-0.935, 0.645)	0.719
DPs	0.268 (-0.457, 0.993)	0.468	-0.432 (-1.860, 0.996)	0.553

**Table 7:** Univariate analysis of maternal factors with neonatal outcomes (imputed dataset) A Comparisons were made with a generalized linear model with the Gaussian family for continuous variables and binomial for binary variables. Results are expressed as coefficients for each variable obtained in the univariate regression model. P-values <0.10 were included in the multivariate model HC: head circumference; pBMI: pre-pregnancy body mass index; DPs: dietary patterns; CI: confidence interval

	Term delivery		Weight Z-score		Height Z-score		HC Z-score	
Maternal factors	Coefficient (95% CI)	P-value	Coefficient (95% CI)	P-value	Coefficient (95% CI)	P-value	Coefficient (95% CI)	P-value
Age	0.09 (0.03, 0.18)	0.04	0.002 (-0.02, 0.02)	0.8]	0.009 (-0.02, 0.03)	0.54	-0.01 (-0.04, 0.01)	0.41
Pre-pregnancy weight	-0.00 (-0.02, 0.02)	0.84	0.001 (0.002, 0.017)	0.003	0.003 (-0.004, 0.11)	0.35	0.007 (0.001, 0.15)	0.08
pBMI classification	0.27 (-0.52, 1.07)	0.50	-0.03 (-0.26, -0.19)	0.75	-0.014 (-0.02, 0.25)	0.91	-0.02 (-0.31, 0.25)	0.84
Education level	-0.08 (-0.86, 0.69)	0.83	-0.08 (-0.31, 0.15)	0.49	0.025 (-0.02, 0.29)	0.84	-0.22 (-0.49, 0.04)	0.10
DPs	-0.11 (-1.47, 1.25)	0.87	-0.07 (-0.36, 0.21)	0.62	0.105 (-0.26, 0.47)	0.58	-0.113 (-0.43, 0.20)	0.49

**Table 8:** Univariate analysis of maternal factors with neonatal outcomes (imputed dataset) B Comparisons were made with a generalized linear model with the Gaussian family for continuous variables and binomial for binary variables. Results are expressed as coefficients for each variable obtained in the univariate regression model. P-values <0.10 were included in the multivariate model NICU: neonatal intensive care unit; pBMI: pre-pregnancy body mass index; DPs: dietary patterns; CI: confidence interval

	Apgar at one minute		Apgar at five minutes		Newborn complications		NICU admission		Length of hospital stay	
Maternal factors	Coefficient (95% CI)	P-value	Coefficient (95% CI)	P-value	Coefficient (95% CI)	P-value	Coefficient (95% CI)	P-value	Coefficient (95% CI)	P-value
Age	0.03 (0.001, 0.06)	0.04	0.01 (-0.007, 0.03)	0.21	-0.06 (-0.23, 0.09)	0.47	-0.05 (-0.23, 0.12)	0.50	-0.42 (-0.72, -0.12)	0.005
Pre-pregnancy weight	0.006 (-0.007, 0.008)	0.87	0.001 (-0.004, 0.007)	0.68	0.05 (0.01, 0.09)	<0.005	0.06 (0.02, 0.10)	0.002	-0.03 (-0.1, 0.05)	0.48
pBMI classification	-0.05 (-0.37, 0.25)	0.70	-0.01 (-0.23, 0.21)	0.90	1.02 (-1.10, 3.16)	0.34	-0.31 (-1.05. 0.42)	0.40	-1.47 (-4.4, 1.4)	0.32
Education level	0.067 (-0.20, 0.33)	0.62	0.01 (-0.18, 0.21)	0.87	0.87 (-0.77, 2.53)	0.29	-0.71 (-2.4, 0.9)	0.41	-0.50 (-3.2, 2.2)	0.71
DPs	0.17 (-0.20, 0.54)	0.36	0.15 (-0.10, 0.42)	0.24	-0.41 (-2.83, 2.01)	0.73	0.68 (-1.76, 3.13)	0.57	0.77 (-1.9, 3.4)	0.57

Multivariate Analysis of Maternal Factors With Pregnancy and Neonatal Outcomes (in the Imputed Dataset)

None of the maternal risk factors was significantly associated with the mode of delivery or maternal complication (Table 9 and Table 10). After correcting for confounders, only maternal age was significantly associated with the one-minute Apgar score and the duration of neonatal stay in the hospital, while pre-pregnancy weight was associated with the neonatal weight Z-score, neonatal complications, and admission to the neonatal intensive care unit (Table 11).

**Table 9:** Multivariate analysis of maternal factors with pregnancy outcomes (imputed dataset) Comparisons were made with a generalized linear model with the Gaussian family for continuous variables and binomial for binary variables. Results are expressed as coefficients for each variable obtained in the univariate regression model. For each outcome, only the explanatory variables significant with a p-value ≤0.10 in the univariate model were included in the multivariate analysis NA: not applicable as variables with p>0.10 in the univariate analysis were not included in the multivariate analysis; pBMI: pre-pregnancy body mass index; DPs: dietary patterns; CI: confidence interval

	Maternal and pregnancy outcomes			
	Mode of delivery		Maternal complications	
Maternal factors	Coefficient (95% CI)	P-value	Coefficient (95% CI)	P-value
Maternal age	NA	NA	NA	NA
Pre-pregnancy weight	0.012 (-0.008, 0.033)	0.254	0.020 (-0.002, 0.044)	0.081
pBMI classification	0.219 (-0.491, 0.930)	0.545	NA	NA
Educational level	NA	NA	NA	NA
DPs	NA	NA	NA	NA

**Table 10:** Multivariate analysis of maternal factors with neonatal outcomes (imputed dataset) A Comparisons were made with a generalized linear model with the Gaussian family for continuous variables and binomial for binary variables. Results are expressed as coefficients for each variable obtained in the univariate regression model. For each outcome, only the explanatory variables significant with a p-value ≤0.10 in the univariate model were included in the multivariate analysis NA: not applicable as variables with p>0.10 in the univariate analysis were not included in the multivariate analysis; HC: head circumference; pBMI: pre-pregnancy body mass index; CI: confidence interval

	Neonatal outcomes						
	Term delivery		Weight Z-score		HC Z-score		
Maternal risk factors	Coefficient (95% CI)	P-value	Coefficient (95% CI)	P-value	Coefficient (95% CI)		P-value
Maternal age (years)	-0.0095 (-0.402, -0.021)	0.541	NA	NA		-0.0095 (-0.402, -0.021)	0.541
Pre-pregnancy weight (kg)	NA	NA	0.00106 (0.0026, 0.017)	0.003	NA		NA
pBMI classification	NA	NA	NA	NA		NA	NA
Educational level	NA	NA	NA	NA		-0.009 (-0.483, 0.059)	0.125

**Table 11:** Multivariate analysis of maternal factors with neonatal outcomes (imputed dataset) B Comparisons were made with a generalized linear model with the Gaussian family for continuous variables and binomial for binary variables. Results are expressed as coefficients for each variable obtained in the univariate regression model. For each outcome, only the explanatory variables significant with a p-value ≤0.10 in the univariate model were included in the multivariate analysis NA: not applicable as variables with p>0.10 in the univariate analysis were not included in the multivariate analysis; pBMI: pre-pregnancy body mass index; NICU: neonatal intensive care unit; CI: confidence interval

	Neonatal outcomes									
Maternal risk factors	Apgar score at one minute			Neonatal complications		NICU admission			Duration of hospitalization	
	Coefficient (95% CI	P-value		Coefficient (95% CI)	P-value	Coefficient (95% CI)	P-value		Coefficient (95% CI)	P-value
Maternal age (years)	0.0307 (0.0014, 0.060)	0.040		NA	NA	NA	NA		-0.427 (-0.725, -0.128)	0.005
Pre-pregnancy weight (kg)	NA		NA	0.054 (0.016, 0.092)	<0.005	0.066 (0.023, 0.1099)	0.002		NA	NA
pBMI classification	NA		NA	NA	NA	NA	NA	NA		NA
Educational level	NA		NA	NA	NA	NA	NA	NA		NA

## Discussion

There was no significant difference in DPs among maternal pBMI groups nor in pregnancy or neonatal outcomes, possibly related to unmeasured confounders, such as maternal exercise, detailed quantitative and qualitative analysis of macronutrient and micronutrient intake, and socioeconomic, genetic, or environmental factors.

DPs are culture-sensitive and population-specific [25]. They help capture the complexities of a diet, and the tools assessing nutrient intake should therefore be culturally adapted.

Based on the cultural type of consumed food by the general Emirati population [26], we identified two distinct DPs emerging in pregnant women: foods with natural ingredients (DP 1) versus processed foods (DP 2). DP 1 includes healthier food components than DP 2 and includes fruits, vegetables, meat or chicken with bread, rice, falafel, whole grains, fish and seafood, olive oil, nuts, seeds, and fat-free or low-fat dairy.

Our results differ from those of a previous study among pregnant Emirati women [13]. Reasons include that the study grouped the DPs into a "Diverse" and a "Western" pattern which are dissimilar to our classification. In addition, that study looked into the dietary effects on gestational weight gain and gestational weight rate instead of pregnancy and neonatal outcomes.

We found no significant difference in the DPs among the maternal pBMI groups nor in pregnancy or neonatal outcomes, similar to previous studies from different countries, strengthening therefore our results [5,6,28,29]. However, our results are in contrast with those of a meta-analysis showing that dietary interventions are effective in improving pregnancy outcomes [9]. The reason for this difference relates perhaps to the heterogeneity of the studies analyzed in that report.

It is possible that the lack of effect on the study outcomes may have resulted from prior health education programs, different food consumption between younger and older pregnant women, or perhaps other unmeasured factors such as genetic predisposition or physical activity [9]. It is also possible that during pregnancy, women with higher pBMI became more motivated than others to adopt a healthier food pattern, to mitigate the effects of excess weight on pregnancy outcomes.

The study has some limitations shared by most surveys by interview. Used in isolation from other dietary methods, FFQ has some limitations [25,30,31]. Superimposed contributors to FFQ weaknesses involve factors influenced by the participants. These include recall bias of foods consumed, social desirability, and the wish to please the researcher, as well as providing information to avoid blame or criticism. Another limitation of this survey is the exclusion of frequently consumed ethnic food (Chinese Indian, etc.). In addition, some participants did not agree to share some data with the research team. Unfortunately, the socioeconomic and educational levels of the participants were not collected as most were reluctant to share such sensitive information. Other limitations were related to the missing values although this was mitigated by the robust statistical multiple imputation method designed to replace them. There is also a limitation inherent to the use of the PCA and the subjectivity in the grouping of the food items, as well as the decision on the number of factors to retain, and the naming of the patterns. Such subjective labeling of the DPs by the research team may also add limitations. This is because, although the selection of those food groupings was in accordance with other international studies, some DPs with similar food content have an alternative designation in other publications [6,13,27]. Furthermore, as the recruitment was done exclusively in selected hospitals within Al Ain, the results are not a representation of pregnant women throughout the UAE, and as a result, our findings cannot be generalized. Our inclusion criteria preclude the generalization of our results to non-high-risk or obese women. Other potential limitations, due to the study design, result from testing the association of DPs with outcomes, but cannot determine causality. We also acknowledge that FFQs reflect the habitual diet over a time period more and cannot predict the exact composition of nutrients which the 24-hour dietary recalls and analysis of consumed nutrients would do [30]. In addition, there was no classification of the participants among the different regions in the city nor according to their urban or rural settings.

Strengths of the study include the efforts exerted to train the field workers on the use of standard interviewing techniques and avoid leading questions. We also used a validated culturally adapted FFQ, created and validated in the city of Al Ain by an experienced team of nutritionists which contributed to the strengths of our results [25]. Furthermore, instead of using birth weight, height, and head circumference at birth as outcomes, we relied instead on their Z-scores as standardized values by gestational age for robust analysis [32,33].

With the ongoing increase in the rate of obesity and the enduring changes in the dietary habits in our population, a periodical review of their resulting impact on pregnancy and neonatal outcomes is required to inform public health policies. Future research should be conducted on a larger scale, on a national level, and applying adequate sampling strategies, with the inclusion of the participants' socioeconomic level, 24-hour dietary recalls, and analysis of the consumed nutrients. As most diet quality indices were developed using dietary guidelines, recommendations, and dietary intake data from high-income countries, it is advisable to develop and validate pregnancy-specific design quality indicators (DQIs) to reflect the nutrition guidelines for culturally diverse countries [34].

## Conclusions

We found no significant difference in DPs among maternal pBMI groups nor a significant impact on pregnancy or neonatal outcomes. This may possibly be attributed to unmeasured confounders, such as maternal exercise, detailed quantitative and qualitative analysis of macronutrient and micronutrient intake, and socioeconomic, genetic, or environmental factors. However, with the increase in the rate of obesity and the changes in the dietary habits in our population, a periodical review of their resulting impact on pregnancy and neonatal outcomes is required to inform public health policies associating FFQs to 24-hour dietary recalls and analysis of the consumed nutrients.

## Figures and Tables

**Figure 2:**
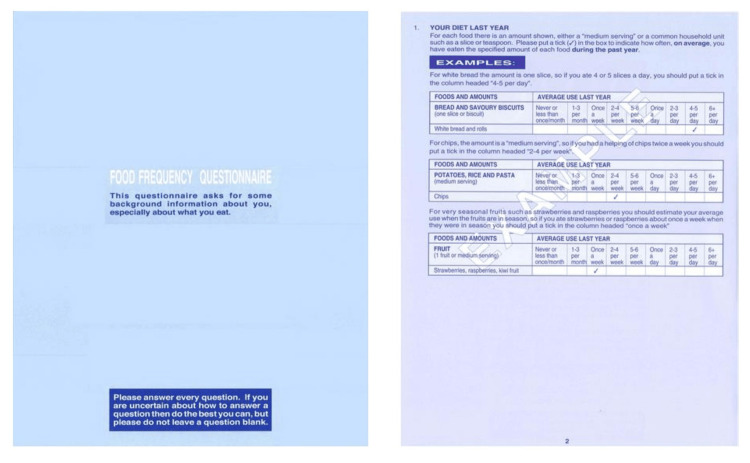
Food frequency questionnaire: English version

**Figure 3:**
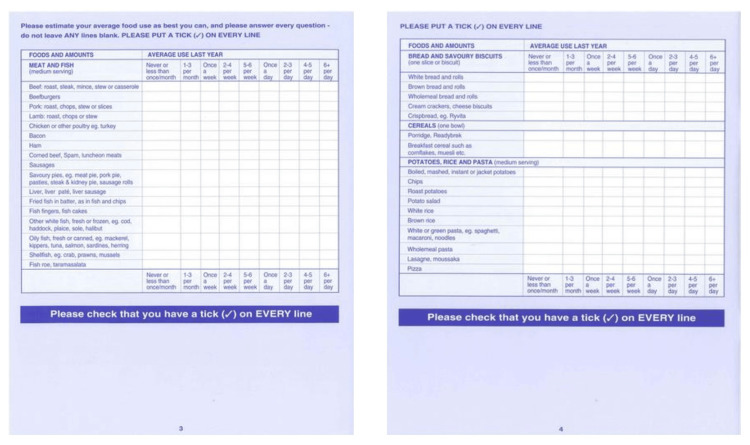
Food frequency questionnaire: English version (Cont.)

**Figure 4:**
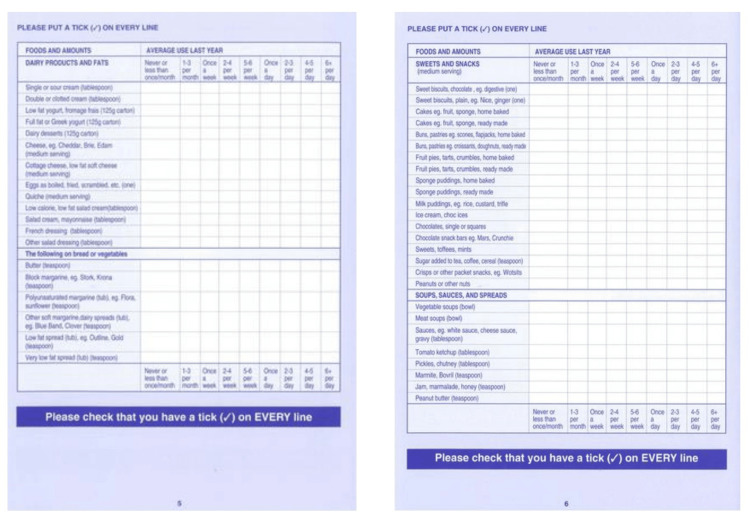
Food frequency questionnaire: English version (Cont.)

**Figure 5:**
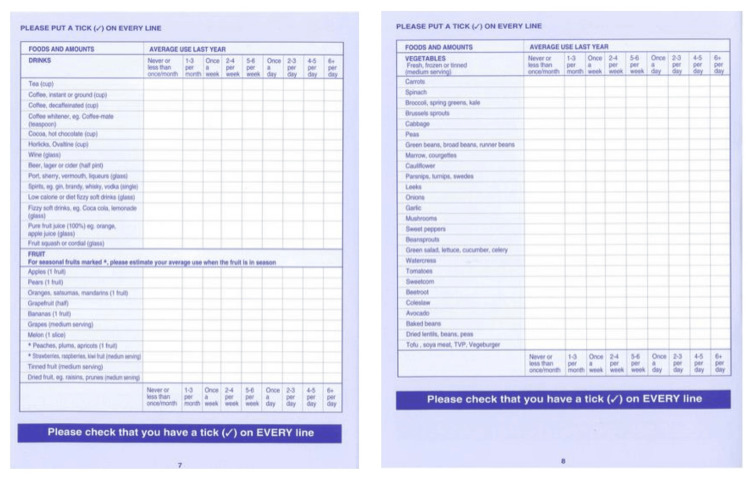
Food frequency questionnaire: English version (Cont.)

**Figure 6:**
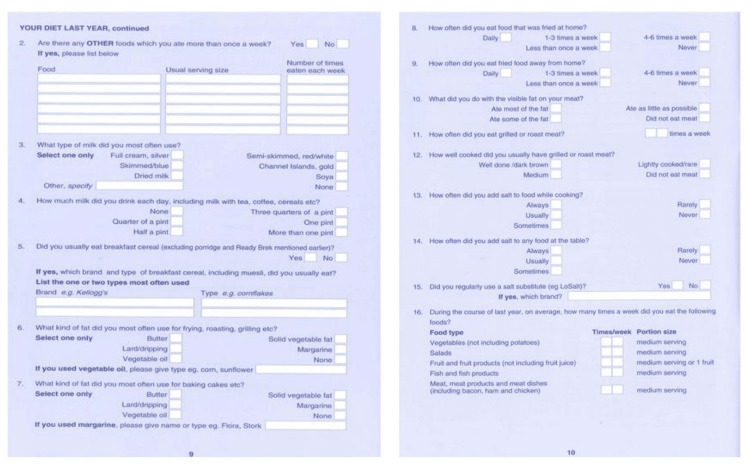
Food frequency questionnaire: English version (Cont.)

**Figure 7:**
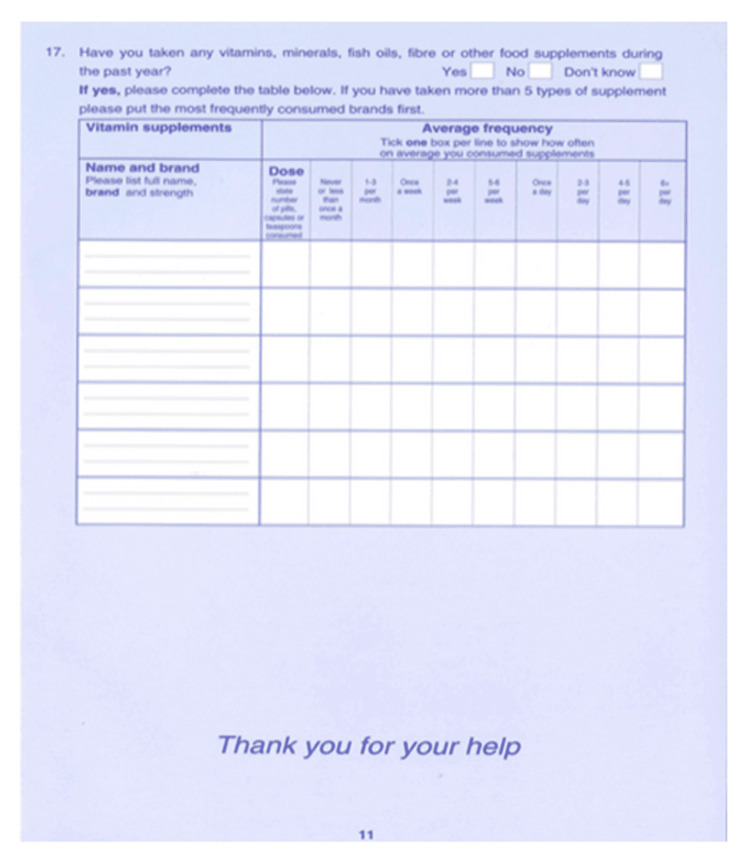
Food frequency questionnaire: English version (Cont.)

## Appendices

Appendix A

**Table 12:** Food frequency questionnaire: Arabic version

مقياس تواتر(تكرار)الطعام
يطلب هذا الاستبيان بعض المعلومات الأساسية عنك، خاصة عن ما تأكله

الخبز و البسكويت المالح										
6 مرات باليوم أو أكثر	4-5 مرات باليوم	2-3 مرات باليوم	مرة واحدة باليوم	5-6 مرات بالأسبوع	2-4 مرات بالأسبوع	مرة واحدة بالأسبوع	3-1 مرات بالشهر	مطلقا أو أقل من مرة بالشهر	الحصة	
	ü									خبز أبيض (سلايس (

معدل الاستخدام خلال العام الماضي									الصنف	
6 مرات باليوم أو أكثر	5-4 مرات باليوم	3-2 مرات باليوم	مرة واحدة باليوم	6-5 مرات بالأسبوع	4-2 مرات بالأسبوع	مرة واحدة بالأسبوع	3-1 مرات بالشهر	مطلقا أو أقل من مرة بالشهر	منتجات الألبان	
									حليب بقر؛ كامل الدسم (كوب)	1
									حليب بقر؛ قليل أو خالي الدسم (كوب)	2
									روب؛ كامل الدسم (علبة 170غم)	3
									روب؛ قليل أو خالي الدسم (علبة 170غم)	4
									لبن آب (كوب)	5
									لبن؛ كامل، قليل أو خالي الدسم (كوب)	6
									شرائح الجبن للسندويشات أو شرائح جبن الشيدر أو شرائح موزاريلا ( شريحة بمقدار اصبعين)	7
									جبنة حلومي أو فيتا ؛ كاملة، قليلة أو خالية الدسم (شريحة بمقدار اصبعين)	8
6 مرات باليوم أو أكثر	5-4 مرات باليوم	3-2 مرات باليوم	مرة واحدة باليوم	6-5 مرات بالأسبوع	4-2 مرات بالأسبوع	مرة واحدة بالأسبوع	3-1 مرات بالشهر	مطلقا أو أقل من مرة بالشهر	الأكلات الشعبية	
									متبل الباذنجان	9
									تبولة ( كوب)	10
									حمص بطحينة	11
									محشي ورق عنب (4 حبات صغار)	12
									محشي كوسا	13
									محشي ملفوف	14
									هريس( لحم أو دجاج ) جريش أو عرسيه (كوب)	15
									(مرقوقة أو ثريد (لحم أو دجاج	16
									صالونة (حدد فقط كمية المرق بالصالونة (اللحم، الدجاج أو السمك	17
									أرز مطبوخ على شكل برياني أو مجبوس أو كبسة أو مندي؛ باستثناء الأرز الأبيض ( صحن متوسط)	18
									معكرونة بصلصة البشاميل و الدجاج ( قطعة بمقدار كف اليد )	19
6 مرات باليوم أو أكثر	5-4 مرات باليوم	3-2 مرات باليوم	مرة واحدة باليوم	6-5 مرات بالأسبوع	4-2 مرات بالأسبوع	مرة واحدة بالأسبوع	3-1 مرات بالشهر	مطلقا أو أقل من مرة بالشهر	بروتينات؛ بيض٬ لحم٬ سمك، فاصوليا واللحوم المصنعة	
									فاصوليا مطبوخة (مثال الفول المدمس)، أو العدس، أو الدانجو(الغير مضاف بالحمص (بطحينة أو بشوربة فاصوليا)	20
									بيض مغلي أو مقلي أو مخفوق (بيضة واحدة)	21
									لحم الغنم أو البقر المطبوخ مع العيش أو الصالونة أو المرقوقة أو الثريد ( قطعة بمقدار كف اليد )	22
									لحم الغنم أو البقر المشوي بالفرن أو على الفحم (المرافق للعيش أو الخبز)؛ مثال الكباب، لحم تكة، شيش طاووق. (باستثناء الهمبورجر أو الشوارما)	23
									لحم الإبل مع العيش أو الصالونة أو المرقوقة أو الثريد	24
									دجاج مطبوخ مع العيش أو الصالونة أو المرقوقة أو الثريد	25
									دجاج مشوي بالفرن أو على الفحم (المرافق للعيش أو الخبز)؛ مثال الكباب، دجاج تكة، شيش طاووق. (باستثناء (الهمبورجر أو الشوارما	26
									(دجاج مقلي بالزيت (مثال دجاج كنتاكي	27
									كبدة، قلب، كلي، طحال	28
									سمك مقلي أو مخبوز؛ المرافق للعيش أو الخبز	29
									سمك أبيض غير مقلي (كسمك الهامور، كنعد، صافي، شعري) المطبوخ مع العيش أو الصالونة	30
									سمك أبيض مشوي بالفرن أو على الفحم كسمك الهامور، كنعد، شعري (المرافق للعيش أو الخبز)	31
									سمك دهني (غير أبيض)، طازج أو معلب (كسمك التونة، الماكريل، السلمون،السردين)؛ (المرافق للعيش أو الخبز)	32
									فواكه البحر كالربيان، الجمبري، أو المحار؛ مشوي أو مقلي أو مطبوخ مع عيش أو صالونة	33
									(سمك مالح (كنعد، عوال	34
									مرتاديلا أو سلامي أو لانشون أو لحـم مقـدد	35
									هوت دوج، نقانـق	36
6 مرات باليوم أو أكثر	5-4 مرات باليوم	3-2 مرات باليوم	مرة واحدة باليوم	6-5 مرات بالأسبوع	4-2 مرات بالأسبوع	مرة واحدة بالأسبوع	3-1 مرات بالشهر	مطلقا أو أقل من مرة بالشهر	خضروات	
									السلطة الخضراء (الرجاء تحديد حصتك المعتادةـ)	37
									جزر طازج؛ بالحبة أو على شكل سلطة أو مطبوخ على شكل صالونة أو مرقوقة أو ثريد	38
									بطاطا مطبوخة؛ مثال بالسلطة أو بالصالونة أو مع العيش (باستثناء البطاطس المقليّة و رقائق بطاطس الشيبس)	39
									بروكلي أو قرنبيط	40
									ملفوف أو كرنب طازج مثال بسلطة الملفوف أو بالسلطة الخضراء، أو مطبوخ على شكل صالونة (الغير مضاف بمحشي ملفوف)	41
									خيار، مع القشر، طازج؛ بالحبة أو على شكل سلطة	42
									فاصوليا خضراء	43
									كوسا، (باستثناء المحشي كوسا)	44
									ورقيات طازجة؛ مثال أوراق الفجل (الرويد)، الجرجير، البربير، البقل	45
									فلفل حلو طازج؛ بالحبة أو على شكل سلطة أو مطبوخ على شكل صالونة أو مرقوقة أو ثريد	46
									قرع أو بطاطا حلوة	47
									باذنجان مقلي أو مطبوخ (الغير مضاف للمتبل)	48
									بامية	49
									حبوب ذرة مطبوخة بالزبدة أو مع السلطة أو بالصالونة	50
									(خضروات مختلطة (طازجة أو مجمدة	51
6 مرات باليوم أو أكثر	5-4 مرات باليوم	3-2 مرات باليوم	مرة واحدة باليوم	6-5 مرات بالأسبوع	4-2 مرات بالأسبوع	مرة واحدة بالأسبوع	3-1 مرات بالشهر	مطلقا أو أقل من مرة بالشهر	حبوب، أرز، مكرونة ، بطاطا	
									حبوب الافطار غير مغلفة بالسكر (مثال كورنفليكس أو رايس كريسبيس)	52
									حبوب الافطار مغلفة بالسكر (مثال كوكو بوبس أو فروستيز)	53
									حبوب الافطار الكاملة مثال رقائق النخالة أو الميوسلي	54
									أرز أبيض أو أرز أبيض بالشعرية	55
									معكرونة؛ (باستثناء المعكرونة مع صلصة البشاميل)	56
									بطاطس مقليّة	57
									شوفان، مثل شوربة الشوفان	58
									(بيتزا (خضار أو لحم أو دجاج	59
6 مرات باليوم أو أكثر	5-4 مرات باليوم	3-2 مرات باليوم	مرة واحدة باليوم	6-5 مرات بالأسبوع	4-2 مرات بالأسبوع	مرة واحدة بالأسبوع	3-1 مرات بالشهر	مطلقا أو أقل من مرة بالشهر	السّاندوتشات والمقبلات	
									فلافل	60
									(سمبوسة (خضار أو لحم أو دجاج	61
									(عرايس (لحم أو دجاج	62
									فطاير، مناقيش (جبن أو لحم أو زعتر أو (سبانخ	63
									(شوارما (لحم أو دجاج	64
									(هامبورجر (لحم أو دجاج	65
6 مرات باليوم أو أكثر	5-4 مرات باليوم	3-2 مرات باليوم	مرة واحدة باليوم	6-5 مرات بالأسبوع	4-2 مرات بالأسبوع	مرة واحدة بالأسبوع	3-1 مرات بالشهر	مطلقا أو أقل من مرة بالشهر	الخبز و البسكويت المالح	
									(خبز أبيض (سلايس	66
									(خبز اسمر(سلايس	67
									(خبز رقاق (الغير مضاف بالثريد	68
									خبز لبناني أسمر أو أبيض	69
									خبز جباب	70
									خبز سمون، خبز الهوت دوج	71
									(شباتي (بدون زيت	72
									خبز مقلي مثال البراتا أو بوري	73
									(كراكرز أو بسكويت مملحة (مثال سالتين	74
6 مرات باليوم أو أكثر	5-4 مرات باليوم	3-2 مرات باليوم	مرة واحدة باليوم	6-5 مرات بالأسبوع	4-2 مرات بالأسبوع	مرة واحدة بالأسبوع	3-1 مرات بالشهر	مطلقا أو أقل من مرة بالشهر	ما يدهن على الخبز أو يضاف على الخضراوات أو على السلطات أو المشروبات	
									لبنة، جبنة قابلة للذهن (بوك، فلادليفيا أو المثلثات أو كيري)، جبنة بيضاء؛ كاملة أو قليلة الدسم ( ملعقة طعام)	75
									زيتون	76
									سمن ( ملعقة طعام)	77
									زبدة ( ملعقة طعام)	78
									مايونيز أو كريم للسلطة ( ملعقة طعام)	79
									سكر (المضاف للشاي كرك، القهوة، الشاي الأحمر)	80
									حليب مبخر أو مكثف، مثال أبو قوس (المضاف للشاي كرك، القهوة، الشاي الأحمر)	81
									مربّى ( ملعقة طعام)	82
									عسل ( ملعقة طعام)	83
									( ملعقة طعام) (شوكولاتة قابلة للدهن (مثال نوتيلا	84
									دبس التمر أو شيرة	85
									عصير ليمون أصفر أو لومي أخضر	86
									كاتشب أو صلصة طماطم	87
									(صلصة حارة (دقوس أو شطة	88
									مخللات أو شاتني أو اجار	89
6 مرات باليوم أو أكثر	5-4 مرات باليوم	3-2 مرات باليوم	مرة واحدة باليوم	6-5 مرات بالأسبوع	4-2 مرات بالأسبوع	مرة واحدة بالأسبوع	3-1 مرات بالشهر	مطلقا أو أقل من مرة بالشهر	فواكه وفواكه جافة	
									تمر أو رطب (كمية متوسطة)	90
									تفاح أو كمثرى (حبة فاكهة)	91
									موز (حبة فاكهة)	92
									برتقال أو صنطرة (يوسف أفندي) أو جريب فروت (حبة فاكهة)	93
									فراولة أو كرز أو ثمرة العليق أو توت (كمية متوسطة)	94
									أناناس (شريحة)	95
									رمان (كمية متوسطة)	96
									عنب (كمية متوسطة)	97
									كيوي (كمية متوسطة)	98
									برقوق أو خوخ أو مشمش أو تين طازج (حبة فاكهة)	99
									مانجو (حبة فاكهة)	100
									بطيخ أو شمام (شريحة واحدة)	101
6 مرات باليوم أو أكثر	5-4 مرات باليوم	3-2 مرات باليوم	مرة واحدة باليوم	6-5 مرات بالأسبوع	4-2 مرات بالأسبوع	مرة واحدة بالأسبوع	3-1 مرات بالشهر	مطلقا أو أقل من مرة بالشهر	مشروبات	
									مشروبات غازية محلاة (مثال بيبسي أو كوكا كولا، بما في ذلك مشروب ماونتن ديو)	102
									مشروبات غازية "دايت/لايت" (مثال بيبسي أو كوكا كولا)	103
									عصير الفاكهة الطبيعية بنسبة 100% (بدون سكر مضاف)	104
									عصير الفواكه من المركزات، مثال كوكتيل (فواكه مع سكر مضاف)	105
									مشروبات الطاقة، مثال ريد بول	106
									ميلك شيك أو سموثي؛ (مثال ميلك شيك الافوكادو)	107
									شراب بنكهة الفاكهة (مثال التانغ، فيمتو، كابري سن)	108
6 مرات باليوم أو أكثر	5-4 مرات باليوم	3-2 مرات باليوم	مرة واحدة باليوم	6-5 مرات بالأسبوع	4-2 مرات بالأسبوع	مرة واحدة بالأسبوع	3-1 مرات بالشهر	مطلقا أو أقل من مرة بالشهر	الحلوى والوجبات الخفيفة	
									بطاطس شيبس	109
									بذور دوار الشمس المحمص أو بذور القرع المحمص	110
									مكسَّرات مشكَّلة	111
									أم علي	112
									كنافة بالجبنة أو بالكريمة	113
									بلاليط	114
									لقيمات	115
									قرص، مثال قرص مفروك	116
									بقلاوة	117
									معمول التمر	118
									كريم كراميل أو كسترد أو مهلبية	119
									(بسكويت أو كوكيز؛ (مثال دايجستف	120
									الكعكة الإسفنجية أو الكب كيك أو البانكيك	121
									دونات	122
									الكرواسون (زعتر أو جبن أو شوكولاته)، أو لفات القرفة أو الجبن، أو التارتولات بالكريمة أو الفواكه	123
									كيكة بالكريمة	124
									أصابع الشوكولاتة (مثال من باتشي، مارس أو سنيكيرز)، أو سكاكر أو حلوى الكراميل	125
									ايس كريم	126
									فشار	127
6 مرات باليوم أو أكثر	5-4 مرات باليوم	3-2 مرات باليوم	مرة واحدة باليوم	6-5 مرات بالأسبوع	4-2 مرات بالأسبوع	مرة واحدة بالأسبوع	3-1 مرات بالشهر	مطلقا أو أقل من مرة بالشهر	الشوربة	
									شوربة بالخضار أو بلحم أو دجاج	128
									(شوربة فاصوليا (مثال شوربة عدس	129
									(شوربة الاندومي (نودلز مجففة فورية	130

نوع الطعام	الكمية المتناولة	عدد مرات تناول هذا النوع في الأسبوع

العلامة التجارية : كلوقز	النوع : كورن فليكس

نوع الطعام	عدد المرات /الأسبوع	حجم الحصة
خضراوات (لا تشمل البطاطا)		حجة متوسطة
السلطات		حجة متوسطة
الفواكه ومنتجاتها(لا تشمل العصائر)		حجة متوسطة
الأسماك ومنتجاتها		حجة متوسطة
اللحم ومنتجاته		حجة متوسطة

الفيتامينات والمكملات	متوسط التكرار (ضع علامة (√) على مربع واحد لكل خط لعرض المكملات التي استهلكتها بشكل متكرر في المتوسط)									
اسم المنتج وعلامته التجاربة	الجرعة:الرجاء ذكر عدد الحبات او الاقراص او الملاعق	مطلقا أو أقل من مرة بالشهر	1-3 مرات بالشهر	مرة واحدة بالأسبوع	2-4 مرات بالأسبوع	5-6 مرات بالأسبوع	مرة واحدة باليوم	2-3 مرات باليوم	4-5 مرات باليوم	6 مرات باليوم أو أكثر

الفترة	أكثر من ثلاثة أشهر	أقل من ثلاثة أشهر	لم آخذه أبدا			
تكرار الجرعة	مرة واحدة في اليوم	2-4 مرات في الأسبوع	5-6 مرات في الأسبوع	مرة واحدة في الأسبوع	أقل من مرة واحدة في الأسبوع	لم تأخذ أي جرعة

الفترة	أكثر من ثلاثة أشهر	أقل من ثلاثة أشهر	لم آخذه أبدا			
تكرار الجرعة	مرة واحدة في اليوم	2-4 مرات في الأسبوع	5-6 مرات في الأسبوع	مرة واحدة في الأسبوع	أقل من مرة واحدة في الأسبوع	لم تأخذ أي جرعة

Appendix B

Appendix C

**Table 13:** Food frequency questionnaire: food groups and dietary items within each group

Food groups and dietary items within each group	
1. Dairy foods	Cow milk: full-fat, low-fat, or skimmed (1). Yogurt: full-fat, low-fat, or skimmed (2). Laban (buttermilk-based drink) (3). Buttermilk (4). Sandwich cheese: sliced. Cheddar cheese: full-fat or low-fat (5). Feta cheese and halloumi cheese: full-fat or low-fat (6)
2. Composite dishes	Mutabbel (Middle Eastern eggplant-based dip) (7). Tabbouleh (Middle Eastern parsley-based salad) (8). Hummus (chickpeas dip) (9). Stuffed grape leaves (10). Stuffed marrow (11). Stuffed cabbage (12). Harees (Middle Eastern pudding-like crushed wheat kernels) (meat or chicken) (13). Margooga and thareed (soaked bread-based mixed dishes) (14). Salona (traditional stew) (15). Cooked mixed rice (16). Macaroni with bechamel (17)
3. Proteins, including vegetarian and animal sources of proteins and processed meats	Baked beans (18). Eggs (19). Lamb/beef: cooked (20). Lamb/beef: grilled (21). Camel meat: cooked (22). Chicken: cooked (23). Chicken: grilled (24). Chicken: fried (25). Organ meat, e.g., liver (26). Fried fish (27). White fish: cooked (28). Grilled fish (29). Oily fish (30). Seafood: grilled or fried or cooked (31). Malleh fish (salted fish) (32). Mortadella, salami, and luncheon meat (33). Hot dog (34)
4. Vegetables	Carrots (35). Potato (36). Broccoli and cauliflower (37). Cabbage (38). Cucumber (39). Green peas (40). Green beans (41). Marrow (42). Lettuce (43). Green vegetables (44). Tomato: raw (45). Onion: raw (46). Green pepper (47). Pumpkin and sweet potato (48). Eggplant (49). Okra (50). Sweet corn (51). Mixed vegetables (52)
5. Cereals (pasta and other cereals), rice, and starches	Non-sugar-coated cereals (53). Sugar-coated cereals (54). Wholegrain cereals and white rice (55). Pasta (56). French fries (57). Oats (58). Pizza (59)
6. Sandwiches and other baked snacks	Falafel (chickpeas-based deep-fried patties) (60). Sambosa (deep-fried pastry) (61). Pakora (vegetable-based fried snack) (62). Arayes (meat-stuffed pita bread) (63). Fatayer (stuffed or topped pie) (64). Shawarma (Middle Eastern sandwich made with rotisserie meat) (65). Hamburger (meat or chicken) (66)
7. Breads and savory biscuits	White bread: slice (67). Brown bread: slice (68). Rgag bread (69). Arabic bread (70). Chebab bread (Emirati pancake) (71). Samoon bread (hot dog bread or bread roll) (72). Chapati (unleavened flat bread) (73). Fried bread, e.g., paratha or puri (74). Crackers and salted biscuits (75)
8. Spreads on breads, on vegetables, or on salads	Labneh (cheese spread) (76). Ghee (clarified butter) (77). Butter (78). Mayonnaise and salad cream (79). Jam (80). Honey (82). Chocolate spread (83). Date syrup (84). Lemon juice (85). Ketchup or tomato sauce (86). Hot chili sauce (87). Pickles or chutney (88). Olives (89)
9. Soups	Soup of vegetables only (90). Soup with meat or chicken (91). Soup with legumes (92). Instant dehydrated soup (93). Instant noodles soup (94)
10. Fruits and dried fruits	Apple and pear (95). Banana (96). Orange and tangerine (97). Strawberries and blueberries (98). Pineapple (99). Pomegranate (100). Grapes (101). Kiwi (102). Plum or peaches (103). Mango (104). Watermelon and melon (105). Fruit salad (106). Dates (107)
11. Beverages	Soft drinks: sweetened (108). Soft drinks: unsweetened (109). Fruit cocktail: sweetened (110). Fruit juice: unsweetened (111). Energy drinks (112). Milkshakes and smoothies (113). Fruit-flavored drinks (114)
12. Sweets and desserts	Um ali (Egyptian bread pudding) (115). Kunafah (Middle Eastern pastry filled with cheese or cream) (116). Balaleet (Emirati sweet vermicelli and eggs) (117). Lgeimat (Emirati fritters) (118). Qurs (Emirati fried sweetened bread) (119). Omani halwa (Omani sweet jelly dessert) (120). Rahash and halwa (Middle Eastern tahini-based sweet) (121). Baklava (Middle Eastern phyllo pastry with spiced walnuts) (122). Maamoul (Middle Eastern date-filled cookies) (123). Pudding (124). Biscuits or cookies (125). Cakes (126). Donuts (127). Croissants (128). Cakes with cream (129). Chocolate bars (130). Ice cream (131). Potato chips (132). Salted sunflower seeds (133). Mixed nuts (134). Popcorn (135)
13. Foods consumed daily	Water (136). Sugar added to beverages (137). Evaporated milk (138). Salt added at the table (139)

Appendix D

**Table 14:** Factor loadings (F) of consumed food groups by factor analysis Extraction method: factor analysis; rotation method: orthogonal Varimax with Kaiser normalization. Only factors with an eigenvalue >1 and an absolute value >0.4 were retained in the table. Total variance explained 35%. All food groups are considered to have an interpretable association with one of the six factors. §: Factors 4, 5, and 6 had an eigenvalue <1 and were therefore not retained in the analysis of dietary patterns

Consumed food	F1	F2	F3	F4^§^	F5^§^	F6^§^
Hummus (chickpeas dip)					0.4161	
Mutabbel (Middle Eastern eggplant-based dip)						
Tabbouleh (Middle Eastern parsley-based salad)						
Stuffed grape leaves					0.5214	
Stuffed marrow (mahshi koosa)					0.5064	
Stuffed coleslaw (malfouf)					0.5064	
Harees (Middle Eastern pudding-like crushed wheat kernels) (meat or chicken)					0.4424	
Margooga (soaked bread-based mixed dishes)						
Salona (traditional stew)						
Cooked rice as in biryani, machbous, kabsa, mandi (other than white rice)			0.4603			
Macaroni with bechamel and chicken						
Baked beans (as in foul medames) or lentils or broad beans or chickpeas)						
Eggs boiled, fried, or scrambled						
Lamb, mutton, or beef, cooked with rice or salona or margouga			0.4309			
Lamb, mutton, or beef, grilled or barbecued (with bread or rice), as in kebab, meat tikka, shish tawook			0.4781			
Camel meat, cooked with rice or salona or margouga			0.4063			
Chicken or turkey, cooked with rice or salona or margouga			0.6027			
Chicken or turkey, grilled or barbecued (with bread or rice), as in kebab, chicken tikka, shish tawook			0.4394			
Fried chicken (e.g., Kentucky chicken)						
Organs (organ meat, e.g., liver)						
Fried fish or paneed fish (with bread or rice)			0.5759			
Non-fried white fish (e.g., hamour, king fish, emperor, etc.) cooked with rice or salona			0.5729			
Grilled white fish (e.g., hamour, king fish, emperor, etc.) with bread or rice			0.6454			
Oily fish (e.g., tuna, salmon, sardines, etc.), with bread or rice			0.5615			
Seafood (e.g., shrimp)						
Malleh fish (salted fish)			0.4948			
Luncheon meat (mortadella, salami, luncheon meat)						
Hot dog, sausages			0.4863			
Green salad						
Carrots, raw as in salad or cooked as in salona or thareed						
Potato, cooked as in a salad or salona or thareed or rice (other than french fries or chips)						
Broccoli or cauliflower						
Cabbage or kale, raw as in coleslaw salad or cooked as in salona (other than mahshi malfouf)						
Cucumber, with peel, raw (as in green salad or fattoush)		0.4143				
Green beans				0.5754		
Vegetable marrow (other than mahshi koosa)				0.4892		
Green leaves (as in radish leaves, watercress, rocca leaves)						
Green pepper, raw as in salad or cooked as in salona or thareed						0.4465
Pumpkin or sweet potato						
Eggplant, fried or cooked (other than in mutabbel)						
Okra				0.4437		
Sweet corn, in butter or salad or in salona						
Mixed vegetables (fresh or frozen)						
Non-sugar-coated cereals (e.g., Corn Flakes, Rice Krispies)						
Sugar-coated cereals (e.g., Sugar Puffs, Coco Pops, Frosties)	0.4061			0.4658		
Wholegrain cereals such as bran flakes or muesli				0.4345		
White rice, rice with vermicelli						
Pasta, boiled (other than pasta with bechamel)						
French fries						
Oats (as in oats soup)						
Pizza (vegetables, meat, or chicken)						
Falafel (chickpeas-based deep-fried patties)					0.5482	
Sambosa (deep-fried pastry)					0.5127	
Arayes (meat-stuffed pita bread)					0.4876	
Fatayer (stuffed or topped pie)					0.4196	
Shawarma (Middle Eastern sandwich made with rotisserie meat)					0.5929	
Hamburger (meat or chicken)					0.4295	
White bread, slice		0.4613				
Brown bread, slice						
Rgag bread (other than in thareed)						
Arabic bread, white or brown		0.5534				
Chebab bread (Emirati pancake)		0.4133				
Hot dog bread or bread roll		0.4133				
Unleavened flat bread		0.4031				
Fried bread (e.g., paratha or puri)						
Crackers and salted biscuits (e.g., saltine crackers)						
Spreads (labneh or cheese spread, e.g., Philadelphia, Triangle, or Kiri) or white cheese, full-fat or low-fat		0.4837				
Olives		0.4585				
Ghee (clarified butter)						
Butter						0.5178
Mayonnaise or salad cream						0.5658
Added sugar (to karak tea, coffee, red tea)						
Evaporated milk (added to karak tea, coffee, red tea)						
Jam						
Honey						
Chocolate spread (e.g., Nutella)						
Date molasses or syrup						
Lemon or lime juice						
Ketchup or tomato sauce						0.6099
Hot chilli sauce, daggous						0.4574
Pickles or chutney						0.4986
Dates		0.448				
Apple or pear		0.6506				
Banana		0.6948				
Orange or tangerine or grapefruit		0.7095				
Strawberries or cherries or blackberries or blueberries		0.6883				
Pineapple		0.4761				
Pomegranate		0.6224				
Grapes		0.6587				
Kiwi		0.5198				
Plum or peach or apricot or fig		0.5737				
Mango		0.613				
Watermelon or melon		0.6808				
Sweetened soft drinks (e.g., Pepsi, Coca-Cola, Mountain Dew)						
Diet or light soft drinks (e.g., Pepsi, Coca-Cola)				0.4812		
Fruit juice (no added sugar), 100% juice						
Fruit cocktail (with added sugar)						
Energy drink (e.g., Red Bull)				0.5915		
Milkshakes or smoothies (e.g., avocado milkshake)				0.5045		
Fruit-flavored drinks (e.g., Tang, Vimto, Capri-Sun)	0.4456			0.4012		
Potato chips						
Salted sunflower or pumpkin seeds	0.4521					
Mixed nuts						
Um ali (Egyptian bread pudding)	0.6892					
Kunafah (Middle Eastern pastry filled with cheese or cream)	0.6233					
Balaleet (Emirati sweet vermicelli and eggs)	0.6216					
Lgeimat (Emirati fritters)	0.6216					
Qurs (Emirati fried sweetened bread)	0.6186					
Baklava (Middle Eastern phyllo pastry with spiced walnuts)	0.6521					
Maamoul (Middle Eastern date-filled cookies)	0.6408					
Crème caramel, custard, pudding, farni	0.6659					
Biscuits or cookies (e.g., Digestive)	0.5688					
Sponge cake or cupcakes or pancakes	0.6171					
Donuts	0.7593					
Croissants (e.g., zaatar, cheese, chocolate), cinnamon rolls, Danish pastries						
Cream cake	0.6676					
Chocolate bars (Snickers, Mars, etc.) or hard candies or caramel candy	0.5994					
Ice cream						
Popcorn	0.4364					
Soup of vegetables only				0.4144		
Soup with legumes (e.g., lentils)				0.5194		
Instant noodles soup (e.g., Indomie soup)				0.4206		

Appendix E

**Table 15:** Dietary patterns: cluster analysis of food groups Food consumption frequency per week is expressed as mean (standard deviation) *: unpaired t-test

	Dietary pattern group		P-value*
	Group 1	Group 2	
N	60 (42.9%)	80 (57.1%)	
Proteins			
Eggs, boiled, fried, or scrambled	3.433 (2.770)	3.975 (2.536)	0.23
Dairy			
Cow milk, full-fat	4.300 (2.913)	5.263 (2.845)	0.05
Cow milk, low-fat or skimmed	4.117 (2.124)	5.050 (1.728)	0.005
Yoghurt, full-fat, low-fat, or skimmed	4.033 (2.442)	4.237 (2.466)	0.62
Laban	4.167 (2.935)	5.250 (2.799)	0.02
Buttermilk, full-fat, low-fat, or skimmed	5.117 (2.917)	5.900 (2.966)	0.12
Sandwich cheese sliced or cheddar cheese or mozzarella or roumy cheese	4.433 (2.714)	5.025 (2.792)	0.21
Halloumi cheese or feta cheese or akkawi cheese, full-fat or low-fat	3.667 (2.754)	5.075 (3.129)	0.006
Composite			
Hummus	3.233 (2.353)	4.013 (2.646)	0.07
Mutabbel	4.283 (2.140)	4.950 (1.841)	0.05
Tabbouleh	3.217 (2.241)	4.263 (2.540)	0.012
Stuffed grape leaves	3.167 (2.345)	4.213 (3.113)	0.03
Stuffed marrow (mahshi koosa)	3.800 (2.441)	5.625 (2.286)	<0.001
Harees, jareesh, arsiya (meat or chicken)	2.950 (2.127)	3.950 (2.619)	0.016
Margooga, thareed (meat or chicken)	3.850 (2.616)	4.438 (2.614)	0.19
Salona (meat or chicken or fish)	3.550 (2.547)	4.013 (2.631)	0.29
Cooked rice as in biryani, machbous, kabsa, mandi (other than white rice)	4.417 (3.169)	5.112 (2.873)	0.17
Macaroni with bechamel and chicken	3.317 (2.813)	3.638 (2.789)	0.5
Baked beans (as in foul medames) or lentils or broad beans or chickpeas	3.150 (2.523)	3.700 (2.426)	0.19
Proteins			
Eggs, boiled, fried, or scrambled	3.433 (2.770)	3.975 (2.536)	0.23
Lamb, mutton, or beef, cooked with rice or salona or margouga	3.067 (2.254)	3.737 (2.427)	0.09
Lamb, mutton, or beef, grilled or barbecued (with bread or rice)	2.900 (2.384)	3.575 (2.540)	0.11
Camel meat, cooked with rice or salona or margouga	5.233 (3.121)	6.263 (2.675)	0.04
Chicken or turkey, cooked with rice or salona or margouga	3.933 (2.816)	4.312 (2.689)	0.42
Chicken or turkey, grilled or barbecued (with bread or rice)	3.300 (2.465)	3.712 (2.296)	0.31
Fried chicken, e.g., Kentucky chicken	3.317 (2.541)	4.438 (2.500)	0.01
Organ meat, e.g., liver, kidneys, etc.	4.167 (2.156)	5.225 (1.646)	0.001
Fried fish or paneed fish (with bread or rice)	2.967 (2.393)	3.575 (2.469)	0.14
Non-fried white fish (e.g., hamour, king fish, emperor, etc.) cooked with rice or salona	3.883 (2.756)	4.300 (2.711)	0.37
Grilled white fish (e.g., hamour, king fish, emperor, etc.) with bread or rice	3.717 (3.087)	4.100 (2.992)	0.46
Oily fish (e.g., tuna, salmon, sardines, etc.) with bread or rice	4.317 (3.078)	5.438 (3.064)	0.03
Seafood, e.g., shrimp	4.633 (3.130)	6.275 (2.747)	0.001
Malleh fish	5.033 (2.610)	5.713 (2.217)	0.09
Mortadella, salami, luncheon meat	5.317 (3.028)	7.275 (1.961)	<0.001
Hot dog, sausages	5.050 (2.600)	6.162 (1.939)	0.004
Vegetables			
Green salad	4.533 (2.925)	5.362 (2.793)	0.09
Carrots, raw as in salad or cooked as in salona or thareed	4.367 (2.828)	5.338 (2.890)	0.05
Potato, cooked as in a salad or salona or thareed or rice (other than french fries or chips)	3.833 (2.781)	4.388 (2.763)	0.24
Broccoli or cauliflower	3.650 (2.399)	5.263 (2.412)	<0.001
Cabbage or kale, raw as in coleslaw salad or cooked as in salona (other than mahshi malfouf)	4.033 (2.577)	5.550 (2.164)	<0.001
Cucumber, with peel, raw (as in green salad or fattoush)	4.900 (2.868)	4.862 (2.872)	0.93
Green beans	4.367 (2.577)	6.312 (1.755)	<0.001
Marrow (other than mahshi koosa)	4.833 (2.637)	5.812 (2.141)	0.01
Green leaves (as in radish leaves, watercress, rocca leaves)	4.917 (2.901)	5.662 (3.040)	0.14
Green pepper, raw as in salad or cooked as in salona or thareed	4.050 (2.561)	5.000 (2.643)	0.03
Pumpkin or sweet potato	4.733 (2.629)	5.800 (2.302)	0.01
Eggplant, fried or cooked (other than in mutabbel)	3.750 (2.022)	4.588 (2.097)	0.02
Okra	3.717 (2.156)	4.888 (1.916)	<0.001
Sweet corn, in butter or salad or in salona	3.567 (2.567)	4.700 (2.626)	0.01
Mixed vegetables (fresh or frozen)	4.933 (3.151)	5.562 (2.942)	0.22
Carbs			
Non-sugar-coated cereals (e.g., Corn Flakes, Rice Krispies)	3.450 (2.266)	4.000 (2.141)	0.14
Sugar-coated cereals (e.g., Sugar Puffs, Coco Pops, Frosties)	4.967 (2.774)	6.037 (2.022)	0.01
Wholegrain cereals such as bran flakes or muesli	4.750 (2.614)	6.362 (1.585)	<0.001
White rice, rice with vermicelli	4.100 (2.569)	4.575 (2.203)	0.24
Pasta, boiled (other than pasta with bechamel)	2.750 (2.405)	3.700 (2.688)	0.03
French fries	3.083 (2.294)	3.312 (2.509)	0.58
Oats as in oats soup	3.867 (2.734)	4.912 (2.542)	0.02
Pizza (vegetables, meat, or chicken)	2.350 (1.812)	4.075 (2.479)	<0.001
Snacks			
Falafel	2.967 (2.350)	3.987 (2.488)	0.01
Sambosa (vegetables, meat, or chicken)	3.150 (2.049)	3.975 (2.050)	0.02
Arayes (meat or chicken)	3.533 (2.111)	4.600 (1.926)	0.002
Fatayer, manakeesh (cheese, meat, zaatar, or spinach)	2.800 (1.894)	3.013 (1.790)	0.49
Shawarma (meat or chicken)	3.367 (2.558)	4.125 (2.518)	0.08
Hamburger (meat or chicken)	2.917 (2.316)	3.175 (2.310)	0.51
Bread			
White bread, slice	4.533 (3.061)	4.325 (2.633)	0.33
Brown bread, Slice	5.033 (2.905)	5.537 (2.981)	0.31
Rgag bread (other than in thareed)	3.700 (3.005)	4.675 (3.018)	0.06
Arabic Bread, white or brown	4.183 (2.977)	4.400 (2.759)	0.65
Chebab bread	4.050 (3.022)	5.787 (2.941)	<0.001
Samoon	4.05 (3.022)	5.787 (2.941)	<0.001
Chapati (without oil)	4.650 (3.236)	5.412 (2.992)	0.15
Fried bread, e.g., paratha or puri	4.400 (3.147)	4.463 (2.929)	0.9
Crackers and salted biscuits (e.g., saltine crackers)	4.500 (2.879)	6.450 (2.667)	<0.001
Spreads			
Labneh or cheese spread (Philadelphia, Triangle, or Kiri) or white cheese	4.000 (2.538)	4.500 (2.338)	0.23
Olives	3.700 (2.824)	4.062 (2.883)	0.46
Ghee	4.583 (3.004)	4.975 (2.955)	0.44
Butter	3.683 (2.087)	4.500 (2.068)	0.02
Mayonnaise or salad cream	3.667 (2.056)	4.162 (2.108)	0.16
Added sugar (added to karak tea, coffee, red tea)	4.483 (2.994)	5.675 (2.867)	0.018
Evaporated milk (added to karak tea, coffee, red tea)	4.583 (2.848)	5.862 (2.915)	0.01
Jam	4.217 (2.505)	5.050 (2.530)	0.05
Honey	3.667 (2.909)	3.750 (3.034)	0.87
Chocolate spread (e.g., Nutella)	3.950 (2.878)	5.213 (2.984)	0.01
Date molasses or syrup	4.583 (3.004)	6.825 (2.364)	<0.001
Lemon or lime juice	4.150 (2.496)	4.862 (2.535)	0.1
Ketchup or tomato sauce	3.583 (2.493)	4.838 (2.987)	0.01
Hot chilli sauce, daggous	4.383 (2.835)	5.200 (3.066)	0.11
Pickles or chutney	4.667 (2.955)	5.650 (3.007)	0.05
Fruits			
Dates	4.800 (2.857)	5.000 (2.733)	0.67
Apple or pear	4.883 (3.141)	4.075 (3.005)	0.12
Banana	4.167 (2.637)	3.725 (2.501)	0.31
Orange or tangerine or grapefruit	4.550 (2.971)	3.775 (2.501)	0.09
Strawberries or cherries or blackberries or blueberries	4.200 (2.692)	3.888 (2.392)	0.46
Pineapple	4.450 (2.626)	5.675 (2.238)	0.003
Pomegranate	3.933 (2.910)	5.000 (2.942)	0.03
Grapes	3.950 (2.626)	4.150 (2.682)	0.66
Kiwi	3.533 (2.453)	4.588 (2.632)	0.01
Plum or peach or apricot or fig	3.933 (2.524)	5.050 (2.560)	0.01
Mango	3.517 (2.467)	3.362 (2.435)	0.71
Watermelon or melon	3.817 (2.369)	3.750 (2.508)	0.87
Beverages			
Soft drinks (sweetened) (e.g., Pepsi, Coca-Cola, including Mountain Dew)	3.783 (2.478)	4.638 (2.512)	0.04
Soft drinks (diet, light) (e.g., Pepsi, Coca-Cola)	5.333 (2.955)	6.912 (2.274)	<0.001
Fruit juice (no added sugar), 100% juice	3.617 (2.344)	4.450 (2.881)	0.07
Fruit cocktail (with added sugar)	4.250 (2.808)	5.862 (2.722)	<0.001
Energy drinks, e.g., Red Bull	6.017 (2.777)	7.688 (1.298)	<0.001
Milkshakes or smoothies (e.g., avocado milkshake)	4.983 (2.972)	7.450 (1.676)	<0.001
Fruit-flavored drink (e.g., Tang, Vimto, Capri-Sun)	4.817 (3.000)	7.175 (2.085)	<0.001
Snacks and sweets			
Potato chips	3.667 (2.556)	4.175 (2.599)	0.25
Salted sunflower or pumpkin seeds	4.383 (2.835)	6.362 (2.649)	<0.001
Mixed nuts	3.550 (2.494)	4.912 (2.761)	0.003
Um ali	4.850 (3.041)	6.175 (2.773)	0.008
Kunafah (cheese or cream)	4.033 (3.036)	5.938 (2.879)	<0.001
Balaleet	2.967 (2.321)	4.013 (2.660)	0.01
Lgeimat	2.967 (2.321)	4.013 (2.660)	0.01
Qurs	3.333 (2.447)	4.263 (2.623)	0.03
Baklava	3.683 (2.937)	6.600 (2.509)	<0.001
Maamoul date cookies	3.633 (2.774)	5.963 (2.866)	<0.001
Crème caramel, custard, pudding, farni	3.467 (2.825)	6.150 (2.829)	<0.001
Biscuits or cookies (e.g., Digestive)	2.817 (2.071)	4.862 (2.474)	<0.001
Sponge cake or cupcakes or pancakes	3.400 (2.663)	5.162 (3.091)	<0.001
Donuts	2.900 (2.113)	5.537 (2.370)	<0.001
Croissants (e.g., zaatar, cheese, chocolate), cinnamon rolls	2.933 (2.342)	3.575 (2.685)	0.14
Cream cake	3.383 (2.387)	5.638 (2.240)	<0.001
Chocolate bars (Snickers, Mars, etc.) or hard candies or caramel candy	3.417 (2.638)	4.487 (2.899)	0.02
Ice cream	3.717 (2.756)	3.837 (3.000)	0.8
Popcorn	3.600 (2.726)	5.237 (3.062)	0.0013
Soups			
Soup of vegetables only	3.683 (2.528)	4.412 (2.544)	0.09
Soup with legumes (e.g., lentils)	4.300 (2.533)	5.312 (2.411)	0.01
Instant noodles soup (e.g., Indomie soup)	4.133 (2.861)	5.938 (2.848)	<0.001
